# Difference of immune cell infiltration between stable and unstable carotid artery atherosclerosis

**DOI:** 10.1111/jcmm.17018

**Published:** 2021-11-03

**Authors:** Jia Gao, Licheng Shi, Jianhua Gu, Dandan Zhang, Wenjun Wang, Xuanfeng Zhu, Jiannan Liu

**Affiliations:** ^1^ Department of Respiratory Medicine Geriatric Hospital of Nanjing Medical University Nanjing China

**Keywords:** carotid artery atherosclerosis, CIBERSORT, immune infiltration, M2 macrophages, unstable atherosclerosis

## Abstract

Atherosclerotic plaque instability contributes to ischaemic stroke and myocardial infarction. This study is to compare the abundance and difference of immune cell subtypes within unstable atherosclerotic tissues. CIBERSORT was used to speculate the proportions of 22 immune cell types based on a microarray of atherosclerotic carotid artery samples. R software was utilized to illustrate the bar plot, heat map and vioplot. The immune cell landscape in atherosclerosis was diverse, dominated by M2 macrophages, M0 macrophages, resting CD4 memory T cells and CD8 T cells. There was a significant difference in resting CD4 memory T cells (*p* = 0.032), T cells follicular helper (*p* = 0.033), M0 (*p* = 0.047) and M2 macrophages (*p* = 0.012) between stable and unstable atherosclerotic plaques. Compared with stable atherosclerotic plaques, unstable atherosclerotic plaques had a higher percentage of M2 macrophages. Moreover, correlation analysis indicated that the percentage of naïve CD4 T cells was strongly correlated with that of gamma delta T cells (*r* = 0.93, *p* < 0.001), while memory B cells were correlated with plasma cells (*r* = 0.85, *p* < 0.001). In summary, our study explored the abundance and difference of specific immune cell subgroups at unstable plaques, which would aid new immunotherapies for atherosclerosis.

## 1 INTRODUCTION

Atherosclerosis is a systemic pathological process, initiated by fat deposition, endothelial inflammation and immune cell infiltration.^1^, ^2^, ^3^ The atherosclerotic plaques consisted of endothelial cells, smooth muscle cells, fibroblast, macrophages and T cells, which narrowed lumen and caused related clinical symptoms like angina and dizziness.^4^ Although atherosclerosis develops slowly and remains silent for decades, it could be life‐threatening when plaque rupture or erosion happens, leading to ischaemic stroke and myocardial infarction.^5^ Vulnerable plaque is characterized by large necrotic core, thin fibrous cap and spotty calcification.^4^ The cause of plaque rupture or erosion remains incompletely understood although the unstable plaques increase ten‐fold times of the risk of acute coronary syndrome compared with stable plaques.^6^ Therefore, earlier identification of vulnerable plaque and prevention of rupture or erosion is of great significance.^7^

Immune cell infiltration within the vessel wall is closely associated with the initiation and progression of atherosclerosis.^8^ Monocyte/macrophage system participated in engulfing oxidized‐low‐density lipoprotein (ox‐LDL), secreting cytokines and chemokines and interacting with other immune cells.^9^ The polarization of macrophages into M1 and M2 exerts pro‐inflammatory and anti‐inflammatory function respectively.^10^ T cells were accounted for ~40% of immune cells in human atherosclerotic lesions. Among them, Th1 cells and NK cells secrete pro‐inflammatory factors, which disorganize the collagen fibres and promote plaques transition to a vulnerable phenotype, while Treg cells produce transforming growth factor‐β and inhibit Th1 and Th17 cell expansion.^11^, ^12^ Th17 cell is another type of T cell that promotes the formation of thick collagen fibres, contributing to the stability of plaque.^13^ However, the role of Th2, Th9 and CD8+T cells in atherosclerosis remains unclear.^14^ In regard to B cells, B1 cells tend to be pro‐atherogenic and B2 exert an anti‐atherogenic role.^15^ Immune cells participate in the pathogenesis, development and regression of atherosclerosis, so modulating cellular or cytokine‐based immune response could be a novel therapeutic target for treatment of atherosclerosis.^16^

Immune infiltration analysis is a novel tool to estimate the abundances of immune cell subtypes in a mixed cell population based on gene expression data. Profile of immune cell infiltration in atherosclerosis has been previously elucidated.^8^, ^17^ However, it is still unclear about the alteration of immune cell proportion between stable and unstable plaques. In this study, we combined the microarray profiles of stable and unstable atherosclerotic carotid artery tissues from Gene Expression Omnibus (GEO) data sets and applied CIBERSORT method to estimate the abundance of 22 immune cell types within tissues. Further, we discussed their effects on the plaque instability of atherosclerosis.

## 2 MATERIALS AND METHODS

### 2.1 Data acquisition

The microarray data were downloaded from GEO database (http://www.ncbi.nlm.nih.gov/geo/). The GSE28829 data set contained 16 advanced atherosclerotic plaque and 13 early atherosclerotic plaque from human carotid arteries.^18^ The data set was based on GPL570 platform ([HG‐U133_Plus_2], Affymetrix Human Genome U133 Plus 2.0 Array).

### 2.2 Evaluation of atherosclerosis‐infiltrating immune cells

CIBERSORT was used to calculate the fractions of infiltrating immune cells within plaque. CIBERSORT is an analytical tool from the Alizadeh Lab, which estimated the abundances of member cell types in a mixed cell population, using gene expression data.^19^ Briefly, it uses a gene expression signature of 547 marker genes called LM22 (Datasheet S1) to quantify the infiltrated immune cell composition fractions.^20^ Twenty‐two types of immune cells included seven types of T cells, naïve /memory B cells, plasma cells, resting/activated NK cells, monocytes, M0–M2 macrophages, resting/activated dendritic cells, resting/activated mast cells, eosinophils and neutrophils. The algorithm used a default signature matrix at 100 permutations and calculated the *p* value with Monte Carlo sampling.

### 2.3 Data normalization and visualization

All analyses were performed using R version 3.6.1. Principal component analysis (PCA) was performed using the ggplot2 package. The bar plot and the heat map were plotted for showing the proportions of 21 types of immune cells. The correlation heat map was drawn to visualize the correlations of 21 types of infiltrating immune cells by using corrplot packages. The violin plot was plotted to show the abundance differences of 21 types of immune cells between stable and unstable atherosclerotic plaques by using the vioplot package. The differences of cell composition between two groups were compared with Wilcoxon test. *p* < 0.05 was considered significant in this study.

## 3 RESULTS

**FIGURE 1:**
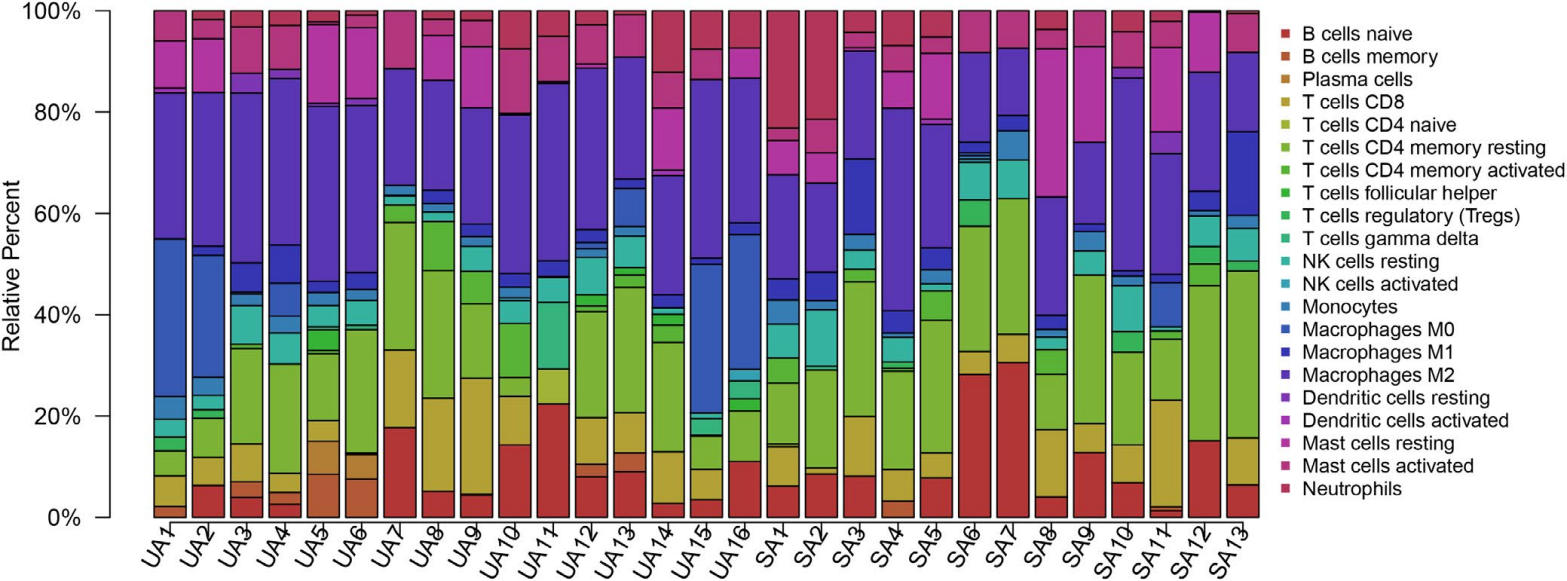
Bar plot of immune infiltration in stable and unstable atherosclerotic carotid artery. Twenty‐two subpopulations of immune cells in 13 stable tissues (SA) and 16 unstable tissues (UA) are shown

**FIGURE 2:**
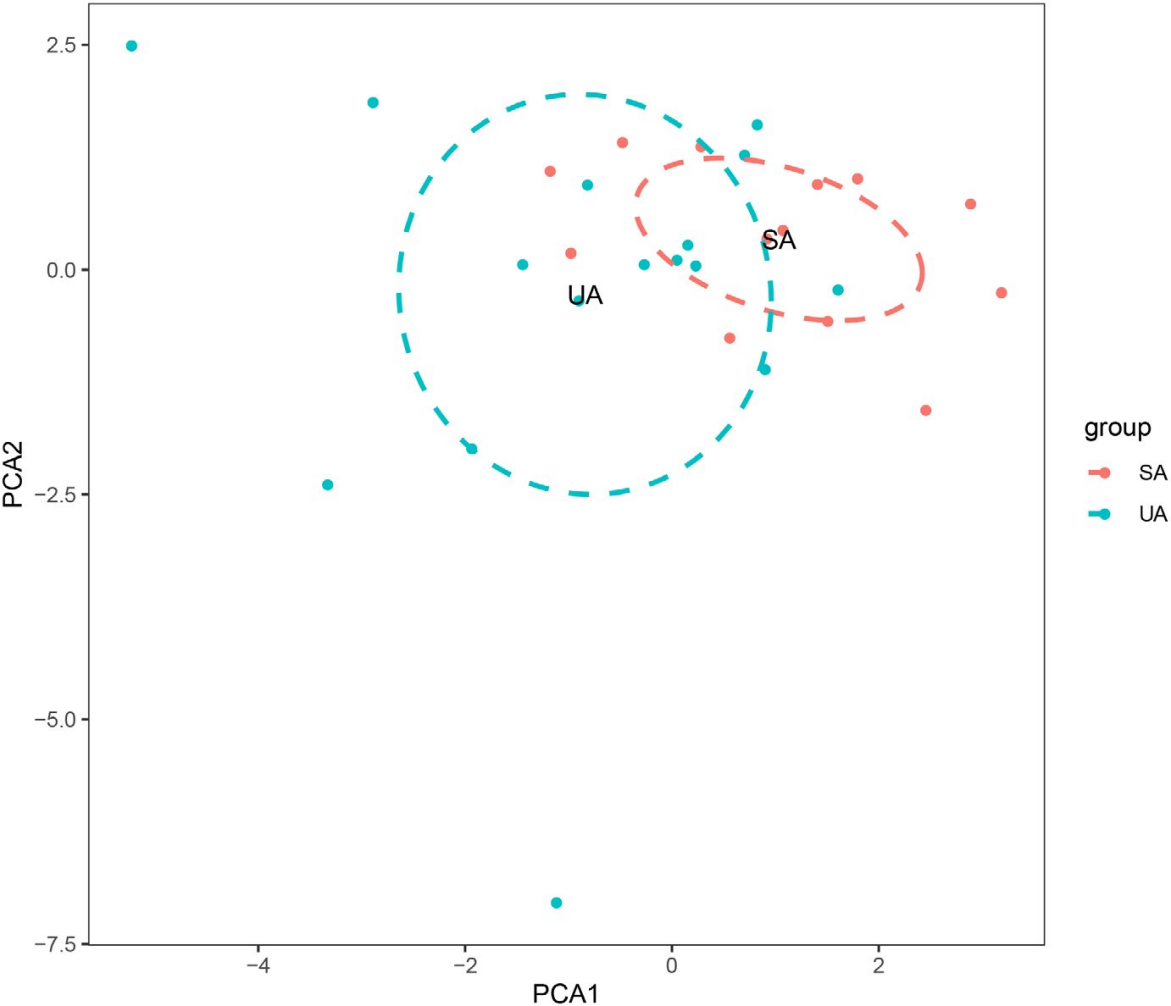
Principal component analysis (PCA) of stable and unstable atherosclerotic arteries. SA: stable atherosclerotic lesions; UA: unstable atherosclerotic lesions

**FIGURE 3:**
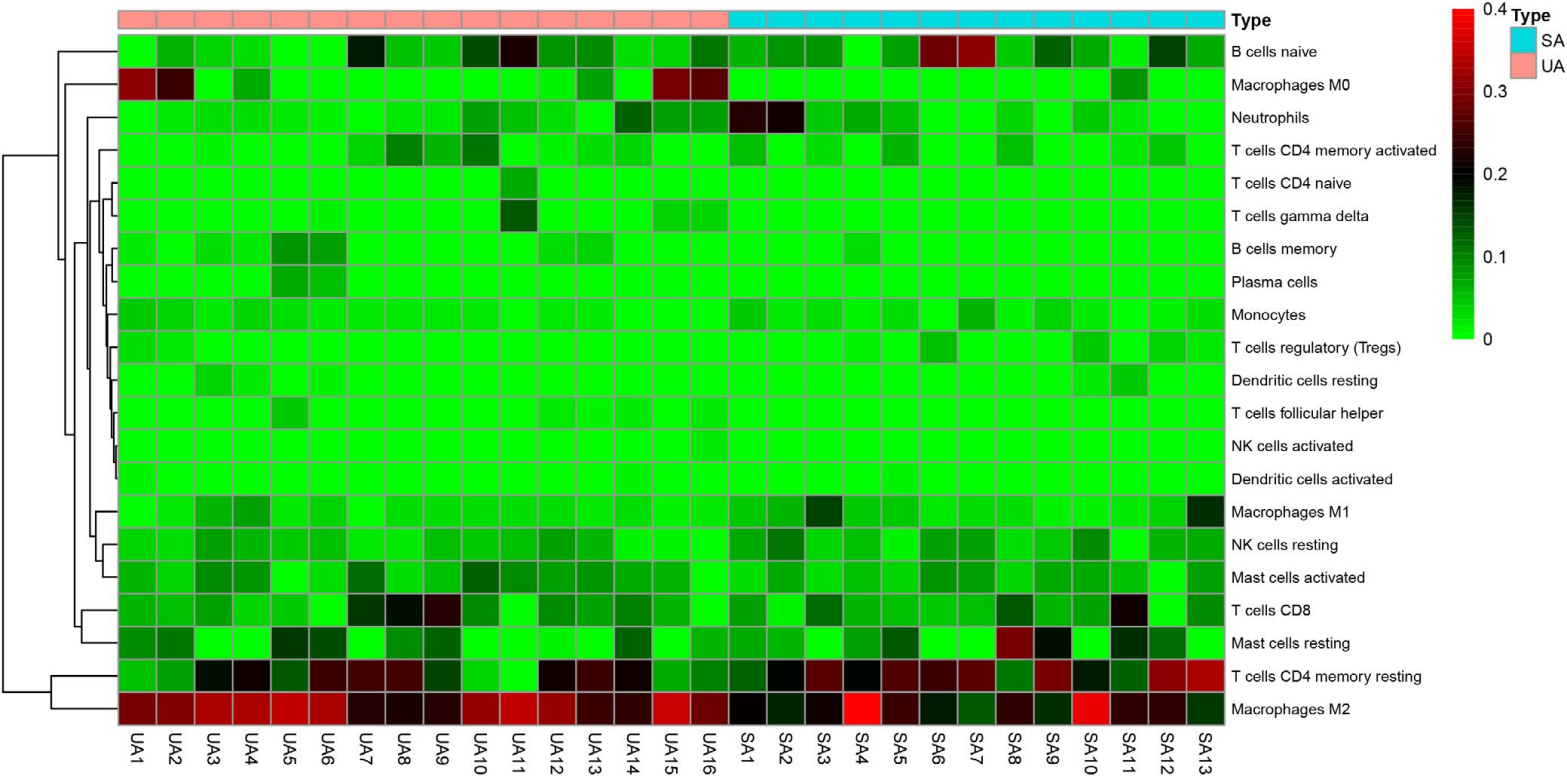
Heat map of 21 immune cell types in the stable and unstable group. Blue represented for stable atherosclerosis, and red represented for unstable atherosclerosis. SA: stable atherosclerotic lesions; UA: unstable atherosclerotic lesions. The colour scale showed the relative percentage of 21 types of immune cells between UA and SA, with red indicating higher abundance and green indicating lower abundance

**FIGURE 4:**
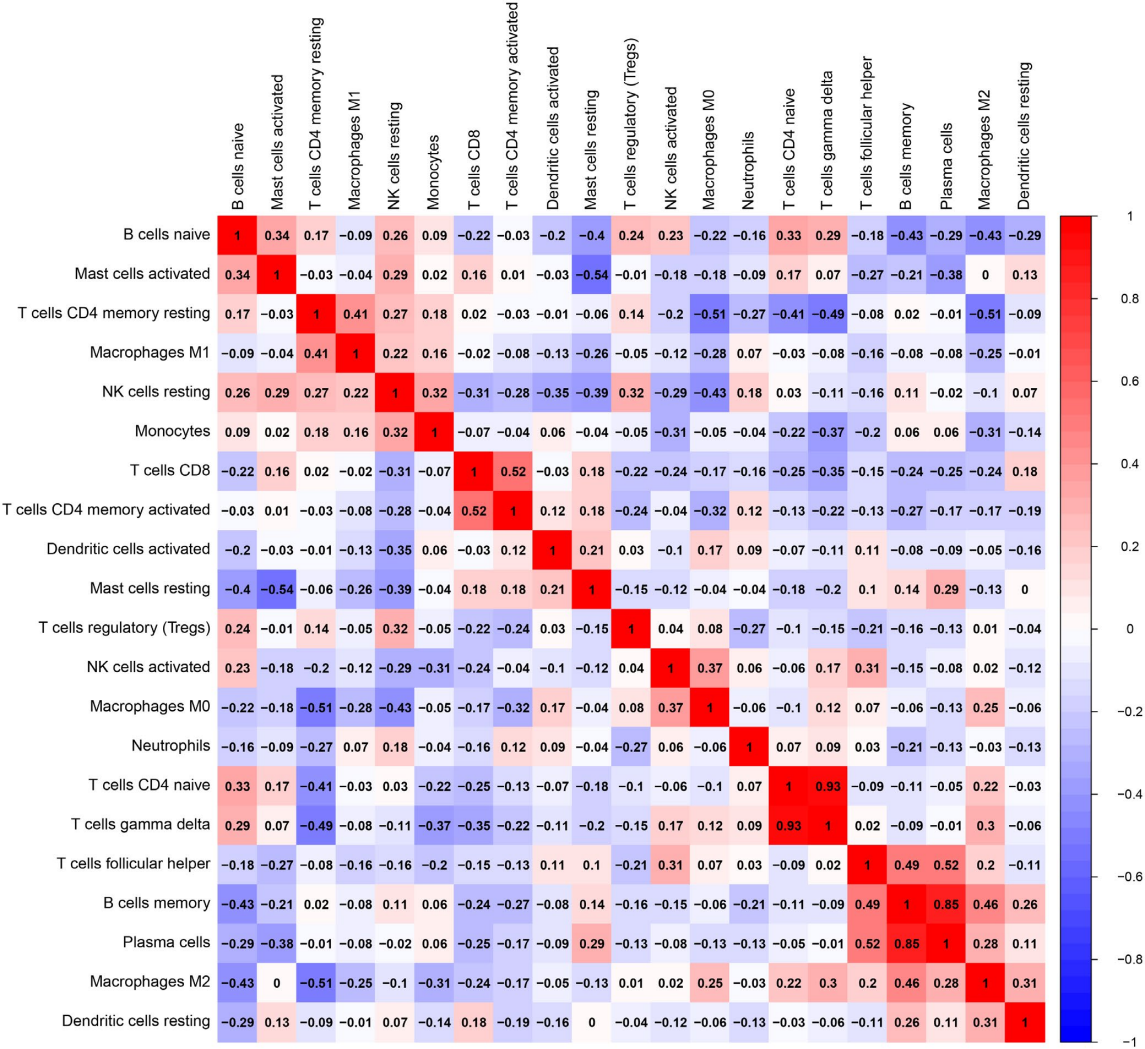
The correlation of immune cell types between stable and unstable atherosclerotic tissues. Red represented positive correlation, and blue represented negative correlation. The *p* value of correlation was shown in Datasheet S1

**FIGURE 5:**
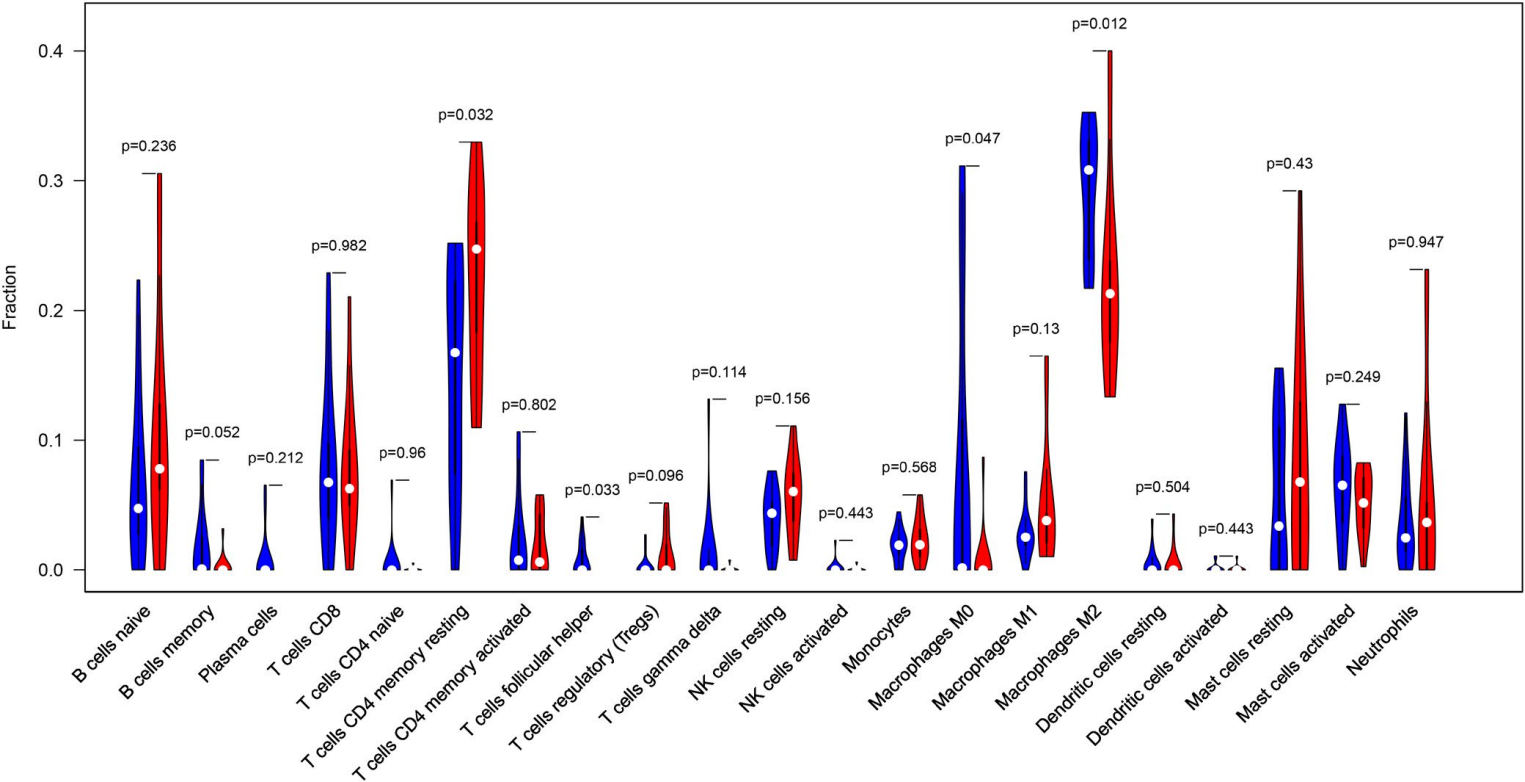
The relative abundance of the immune cells in the two groups. The stable group is marked in blue, and the unstable group is marked in red. *p* values < 0.05 were considered statistically significant

Before performing CIBERSORT analysis, the probe names of microarray were replaced with gene symbols. The CIBERSORT results (Figure 1) showed 21 subpopulations of immune cells (no percentage of eosinophils) in 29 carotid artery samples (13 stable and 16 unstable plaque). The abundance of immune cells from 29 samples showed the obvious group‐bias clustering by the PCA plot (Figure 2). It suggested that difference in immune infiltration was not enough to explain the diversity of samples. The carotid artery samples were separated into two discrete groups based on the clustering of 21 immune cell subpopulations (Figure 3). Besides, we further estimated the correlation between the types of immune cells, and it showed some subpopulations were strongly correlated (Figure 4). Correlation analysis indicated that the percentage of naïve CD4 T cells was strongly correlated with that of gamma delta T cells (*r* = 0.93, *p* < 0.001), while memory B cells were correlated with plasma cells (*r* = 0.85, *p* < 0.001). The *p* value of correlation was shown in Datasheet S2. Compared with stable plaque, unstable plaque had increased infiltration of follicular helper T cells, M0 and M2 macrophages while decreased proportions of resting CD4 memory T cells (Table 1; Figure 5).

**TABLE 1:** The percentage and difference of infiltrating immune cells in unstable and stable atherosclerotic carotid tissues

Immune cell	Stable atherosclerosis	Unstable atherosclerosis	*p* value
B cells naive	10.21609	7.998025	0.12
B cells memory	0.032123	0.350115	0.222
Plasma cells	0	0.048382	‐
T cells CD8	1.542545	0.672867	0.688
T cells CD4 naive	0.802373	0.484355	0.368
T cells CD4 memory resting	22.39444	18.10844	0.107
T cells CD4 memory activated	0.723552	2.11933	0.75
T cells follicular helper	8.893724	7.561705	0.647
T cells regulatory	0.177942	0.488988	0.065
T cells gamma delta	0.227185	0.503963	0.954
NK cells resting	3.152642	5.156772	0.215
NK cells activated	1.861562	0.219522	0.048
Monocytes	7.365122	3.263121	0.375
Macrophages M0	6.238112	7.112336	0.936
Macrophages M1	2.657714	2.453605	0.46
Macrophages M2	0.896512	1.888103	0.799
Dendritic cells resting	10.90528	10.07856	0.128
Dendritic cells activated	9.242971	16.13458	0.095
Mast cells resting	6.671714	4.701726	0.803
Mast cells activated	0	0	‐
Eosinophils	1.638396	4.505282	0.701
Neutrophils	4.359997	6.150221	0.936

## 4 DISCUSSION

Atherosclerosis is a chronic inflammatory vascular disease. Multiple types of immune cells participate in the pathological progression across the whole stages. In our study, M0 and M2 macrophages, resting CD4 memory T cells and CD8 T cells have a dominate abundance within atherosclerotic vessels. Moreover, resting CD4 memory T cells, T cells follicular helper, M0 and M2 macrophages showed a different infiltration percentage between stable and unstable atherosclerotic plaques, which could be an immunotherapy target for atherosclerosis.

By using the CIBERSORT tool, we discovered the differences of 21 immune cells between stable and unstable plaque samples. As it was shown, the majority of immune cells in atherosclerotic tissues were resting CD4 memory T cells, CD 8 T cells, M0 and M2 macrophages, which was consistent with previous results that T cells and macrophages represented the largest population of leukocytes in atherosclerotic plaques.^21^ Unstable plaques had increased infiltration of M0 and M2 macrophages while decreased proportions of resting CD4 memory T cells compared to stable plaque. Depending on the stimuli, M0 macrophages could polarize towards the pro‐inflammatory M1 subset or anti‐inflammatory M2 subsets. These stimuli included cytokines, chemokines, immune complexes and lipids.^22^ In atherosclerotic lesions of Ldlr‐deficient mice, M1 and M2 subsets accounted for 40% and 20% respectively.^23^ Previous report showed that symptomatic plaques displayed fewer pro‐inflammatory genes and more percentage of macrophages with reparative M2 phenotype,^8^ which was in consistent with our results. Increased infiltration of M2 macrophages was atheroprotective and drove atherosclerotic inflammation resolution and plaque regression,^24^ which could be a protective increasement in unstable atherosclerotic plaque.

A strong correlation was also noteworthy between the percentage of naïve CD4 T cells and gamma delta T cells, as well as memory B cells were correlated with plasma cells. CD4+T cells, with the capacity to differentiate into various Th cell or Treg cell subtypes, are commonly found in atherosclerotic plaques and exert different functions.^14^, ^25^, ^26^ Patel et al. reported that Treg cells were increased in unstable plaques,^27^ being related to the stability of plaques both in human and mouse.^28^, ^29^, ^30^, ^31^, ^32^, ^33^ As low number of Treg cells was also detected during the whole stages of human atherosclerotic lesions compared with other chronic inflammation disease^34^ and no statistical difference was found between stable and unstable plaques, our result cannot confirm the relationship between Treg cells and the stability of plaques. Gamma delta T cells are T cells that express a unique T‐cell receptor composed of one γ‐chain and one δ‐chain and found in the gut mucosa, skin and lungs and are involved in the initiation and propagation of immune responses. A clinical study found that a 1‐SD increment in the proportion of gamma delta T cells was associated with 2.4 mmHg higher average systolic blood pressure.^35^ Gut gamma delta T cells were reported to regulate metabolism and accelerate cardiovascular disease.^36^ The role of gamma delta T cells in atherosclerosis was scared and deserved to be further investigations. As atherosclerosis is a complicated, multifactorial pathophysiological process, immune cells in the plaque also interacted and exerted a synergetic role which may explain the correlation between immune cells.^37^, ^38^ However, whether changes of these correlations may affect the stability remained unclear.

Single‐cell proteomic and transcriptomic analyses have been used for uncovering the immune cell landscape in stable and unstable plaques of carotid artery.^8^ Compared with it, we focussed unbiasedly on more immune cell subtypes in a mixed tissue and elucidated the abundance and the correlation between different cells. Our findings also complemented their results. However, some limitations need caution in our study. Firstly, CIBERSORT is insufficient to uncover the whole subtypes in atherosclerosis, and other software could be used, such as EPIC, TIMER and ImmuCellAI. Secondly, this was a bioinformatic analysis, and the predictions need to be confirmed by functional validation in vitro and in vivo.

## 5 CONCLUSION

In summary, utilizing CIBERSORT method, we uncovered the abundance and difference of immune cell types within unstable atherosclerotic carotid artery tissues. Our finding may provide some clues for future immunotherapy in atherosclerosis.

## CONFLICT OF INTEREST

All authors declare that they have no competing interests.

## AUTHOR CONTRIBUTIONS

**Jia Gao:** Formal analysis (equal); writing – original draft (equal). **Licheng Shi:** Formal analysis (equal); visualization (equal). **Jianhua Gu:** Investigation (equal); validation (equal). **Dandan Zhang:** Project administration (equal); software (equal). **Wenjun Wang:** Data curation (equal); resources (equal). **Xuanfeng Zhu:** Conceptualization (equal); writing – review & editing (equal). **Jiannan Liu:** Project administration (equal); software (equal).

## Supporting information

Datasheet S1

DataSheet S2

## Data Availability

The microarray used in our study has been deposited in GEO (GSE28829).
